# Imaging β-Galactosidase Activity in Human Tumor Xenografts and Transgenic Mice Using a Chemiluminescent Substrate

**DOI:** 10.1371/journal.pone.0012024

**Published:** 2010-08-06

**Authors:** Li Liu, Ralph P. Mason

**Affiliations:** Department of Radiology, The University of Texas Southwestern Medical Center, Dallas, Texas, United States of America; University of Texas M. D. Anderson Cancer Center, United States of America

## Abstract

**Background:**

Detection of enzyme activity or transgene expression offers potential insight into developmental biology, disease progression, and potentially personalized medicine. Historically, the *lacZ* gene encoding the enzyme β-galactosidase has been the most common reporter gene and many chromogenic and fluorogenic substrates are well established, but limited to histology or *in vitro* assays. We now present a novel approach for *in vivo* detection of β-galactosidase using optical imaging to detect light emission following administration of the chemiluminescent 1,2-dioxetane substrate Galacto-Light PlusTM.

**Methodology and Principal Findings:**

B-gal activity was visualized in stably transfected human MCF7-*lacZ* tumors growing in mice. LacZ tumors were identified versus contralateral wild type tumors as controls, based on two- to tenfold greater light emission following direct intra tumoral or intravenous administration of reporter substrate. The 1,2-dioxetane substrate is commercially available as a kit for microplate-based assays for β-gal detection, and we have adapted it for *in vivo* application. Typically, 100 µl substrate mixture was administered intravenously and light emission was detected from the *lacZ* tumor immediately with gradual decrease over the next 20 mins. Imaging was also undertaken in transgenic ROSA26 mice following subcutaneous or intravenous injection of substrate mixture.

**Conclusion and Significance:**

Light emission was detectable using standard instrumentation designed for more traditional bioluminescent imaging. Use of 1,2-dioxetane substrates to detect enzyme activity offers a new paradigm for non-invasive biochemistry *in vivo*.

## Introduction

One of the hottest topics is biology today is non-invasive characterization of *in vivo* biochemical processes using various imaging modalities [1], [2]. Detection of enzyme activity or transgene expression *in vivo* offers potential insight into developmental biology, disease progression, and potentially personalized medicine. Historically, the *lacZ* gene encoding the enzyme β-galactosidase (β-gal) has been the most common reporter gene used in molecular biology [3], [4], [5]. Due to its broad spectrum of activity, many chromogenic and fluorogenic substrates are well established, but they are generally limited to histology or *in vitro* assays [6], [7], [8], [9]. Thus, there is an increasing interest in the development of non-invasive reporter techniques to assay *lacZ* gene expression *in vivo*.

Several recent studies have reported novel substrates or novel applications of substrates allowing detection of β-galactosidase *in vivo*. Most current approaches have required direct injection of the substrate into the tissue of interest, *e.g*., photoacoustic tomography (PAT) of 4-chloro-3-bromoindole-galactose (X-gal) [10], single photon emission computed tomography (SPECT) of 5-[I-125]iodoindol-3-yl-β-*D*- galactopyranoside ([I-125]IBDG) [11], and positron emission tomography (PET) of 2-(4-[125I/123I]iodophenyl)ethyl-1-thio-β-*D*-galactopyranoside, 3-(2'-[F-18]fluoroethoxy)-2-nitrophenyl-β-*D*-galactopyranoside or 3-[C-11]methoxy-2-nitrophenyl β-*D*-galactopyranoside [12], [13]. A variety of substrates based on isomers and analogs of 4-fluoro-2-nitrophenyl-β-*D*-galactopyranoside [14], [15], [16], which exhibit ^19^F NMR chemical shift change due to β-gal activity has been presented, demonstrating the ability to differentiate wild type (WT) and stably transfected *lacZ* expressing breast and prostate cells [15], [17] and human tumor xenografts growing in mice [18], [19]. Perhaps the most elegant MRI study to date used a galactose-capped gadolinium ligand (EgadMe) to follow cell lineage in developing tadpoles by ^1^H MRI microscopy following direct intracellular injection of substrate [20]. We have shown the ability to identify *lacZ* versus WT MCF7 tumors in mice using T_2_*-weighted ^1^H MRI following direct intratumoral injection of S-Gal® [21].

In vivo detection of β-gal activity based on systemic administration of reporter molecules has been achieved using a tandem approach based on bioluminescence of Lugal (6-o-β-galactopyranosyl-luciferin) following intraperitoneal (IP) administration [22]. However, this approach requires doubly transfected cells, whereby β-gal (*lacZ* expression) releases luciferin, which becomes a substrate for luciferase. ^1^H MRI signal enhancement was observed in CT26 tumors (wild type versus *lacZ*) growing in mice following intravenous (IV) administration of a gadolinium capped ligand (GD-DOTA-FBG) [23]. The most widely used approach currently exploits fluorescence to detect a 50 nm shift accompanying β-gal activated cleavage of DDAOG (7-hydroxy-9H-(1,3-dichloro-9,9-dimethylacridin-2-one-7-yl) β-*D*-galactopyranoside) revealing β-gal activity in stably transfected human tumors in mice following IV administration [24], [25].

It occurred to us that substrates designed for chemiluminescent imaging (CLI) of enzyme activity using traditional high throughput plate readers could provide an alternative approach to detect *lacZ* gene expression *in vivo*. Detection of emitted light *in vivo* may be considered bioluminescent imaging (BLI), although BLI is often associated with activity of luciferases. We now demonstrate the use of exploiting Galacto-Light PlusTM *in vivo* to detect gene activity in *lacZ* transfected MCF7 tumor cells, MCF7-*lacZ* xenograft tumors, and transgenic *lacZ* gene expressing mice.

## Results

**Figure 1:**
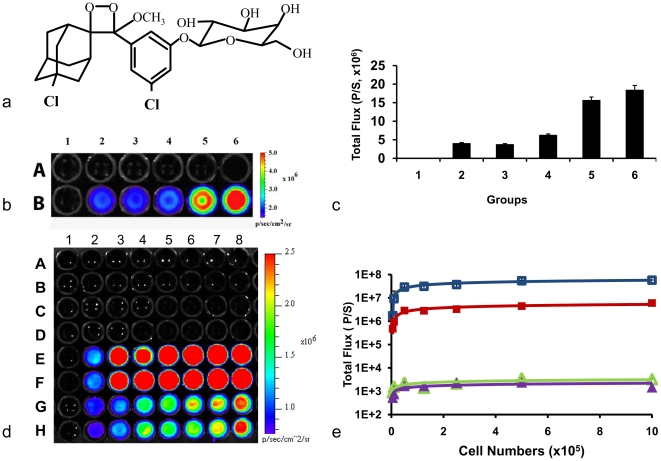
Detection of β-gal activity by chemiluminescent imaging (CLI) using β-gal enzyme and cultured cells. **a**) The chemical structure of Galacto-Light PlusTM substrate; **b**) Differential light emission from wells containing various enzyme + substrate mixtures. Row A: 10 µl PBS; Row B: β-galactosidase (1 U in 10 µl PBS (pH: 7.2-7.4)); **1**: +20 µl PBS; **2**: +1 µl Galacto-plus (diluted to 10 µl in PBS)+10 µl PBS; **3**: +10 µl reaction buffer) +10 µl PBS, **4**: +10 µl accelerator buffer +10 µl PBS; **5**: +1 µl Galacto-plus +10 µl reaction buffer +10 µl accelerator buffer (1∶10∶10); **6**: +2 µl Galacto-plus +8 µl reaction buffer +10 µl accelerator buffer (1∶4∶5) (Total volume: 30 µl per well). **c**) CLI signal intensity for mixtures in (b) **d**) Varying numbers of MCF7-*WT* (upper A&B) and MCF7*-lacZ* (lower C&D) breast cancer cells in wells (0, 1×10^3^, 5×10^3^ ,1×10^4^, 5×10^4^, 1×10^5^, 5×10^5^, 1×10^6^ cells, respectively) imaged using a sensitive CCD camera (exposure time 2 s) following addition of Galacto-Light PlusTM mixture (comprising 10 µl substrate +10 µl accelerant + buffer with (rows A,B E, F) or without (rows C, D, G, H) added lysis buffer); **e**) Signal intensities for MCF7-*lacZ* (▪) and -WT (**Δ**) cells in (d), where open symbols indicate inclusion of lysis buffer. Exposure times ranged from 5 s to 120 s to ensure adequate SNR without overloading.

The Galacto-Light Plus kit includes several components, and the importance of each was tested in solution with enzyme. Substrate (3-chloro-5-(5'-chloro-4-methoxyspiro[1,2-dioxetane-3,2'-tricyclo[3.3.1.13,7]decan]-4-yl)phenyl β-*D*-galacto pyranoside (Galacton Plus), Figure 1a), reaction buffer, and accelerant (EmeraldTM enhancer and diethanolamine in buffer) were tested with β-galactosidase. A faint glow was detected for enzyme plus substrate alone with or without the additional individual reaction and accelerant buffers, but all three together gave substantially higher signal and a ratio of 1∶4∶5 substrate: reaction buffer: accelerant gave the strongest signal (Figure 1b and c). The mixture was applied to various concentrations of MCF7-WT and –*lacZ* cells (Figure 1d and e). Light emission was found to increase with increasing cell numbers, particularly below 50,000 cells, though above this tended to plateau. Light detected from the WT cells was about 10,000 fold less intense. Addition of lysis buffer to cells increased the emitted light by a factor of about 10 for the *lacZ* cells, but had less effect on WT cells (less than two-fold) (Figure 1e). Maximum light emission was found at about 540 nm for a reaction mixture in solution and 530 nm when determined in minced tissue (Figure S1).

**Figure 2:**
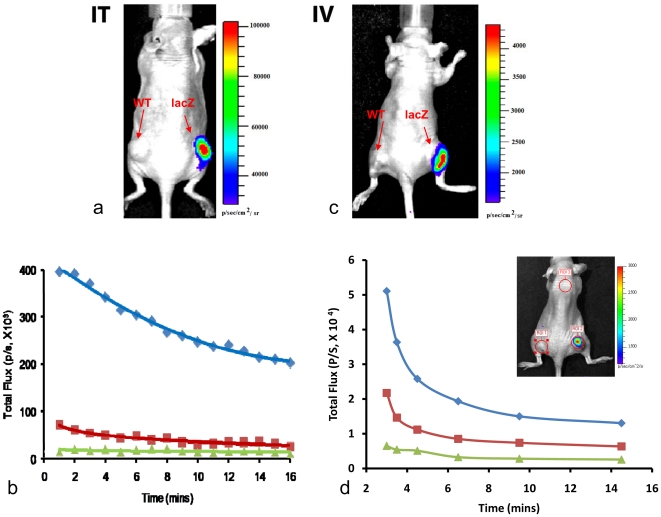
Imaging β-gal activity *in vivo*. **a**) Galacto-Light PlusTM substrate mixture (50 µl) was injected intratumorally (IT) into WT and *lacZ* tumors respectively, revealing the *lacZ* tumor based on light emission with a 10 s exposure time. **b**) Signal dynamics for regions of interest in (a): *lacZ*-tumor (blue), WT tumor (red) and upper back (green). **c**) Optical following IV injection of Galacto-Light PlusTM mixture (100 µl) with 60 s exposure time (relative light emission 5.5 fold higher for *lacZ* vs. WT tumor). **d**) Light emission dynamics for a second tumor-bearing mouse treated as in c. Curves show signal for specific regions of interest (inset) with highest signal from the *lacZ* tumor (blue curve), then contralateral WT tumor (red) and lowest for skin on the upper back (green) each of which decreased with half-life of about 2 mins. Corresponding histology and β-gal expression profiles are shown in Figure S2.

Direct injection of Galacto-Light Plus mixture (30 µl) intratumorally (IT) gave a strong signal in MCF7-*lacZ* tumors easily detectable in 10 s and much less signal in WT tumors (Figure 2a). Typical integrated signal for *lacZ* tumor was 4.0×10^5^ photons/sec, whereas a similarly sized contralateral WT tumor gave 7.1×10^4^ photons/sec, providing over 7-fold contrast, while skin on the back gave about 1.5×10^4^ photons/sec and background noise was only 7x10^3^ photons/sec. Administration of a 50 µl mixture (30 µl substrate + 10 µl accelerant + 10 µl reaction buffer) gave a much higher relative signal (average 10.6 for three tumor pairs) than an alternate mixture (20∶5∶5 µl), which gave average 2.5). In general, the relative signal for *lacZ* versus WT tumor was found to be superior for longer signal acquisition times. A dynamic signal intensity curve showed decrease after 3 mins reaching about 50% after 15 mins (Figure 2b). Following IV injection MCF7-*lacZ* tumor showed signal (SNR 8.6), though it was somewhat less intense than following IT injection. Nonetheless, it was significantly more intense than for WT with a contrast of about five-fold (Figure 2c). In a separate animal, dynamic variation in emitted light was assessed over a period of 15 mins following IV injection (Figure 2d). Intense signal was observed from the *lacZ* tumor with about two-fold less signal from the control WT tumor and a further two-fold less signal from a region of skin on the foreback. A rapid decline in signal was observed at each location with a half-life of about 2 mins at each location. Histology using X-gal and H&E staining together with traditional colorimetric assay and Western blot of tissues confirmed β-gal activity in the MCF7*-lacZ* tumors and about 10-fold less in WT tumors (Figure S2).

**Figure 3:**
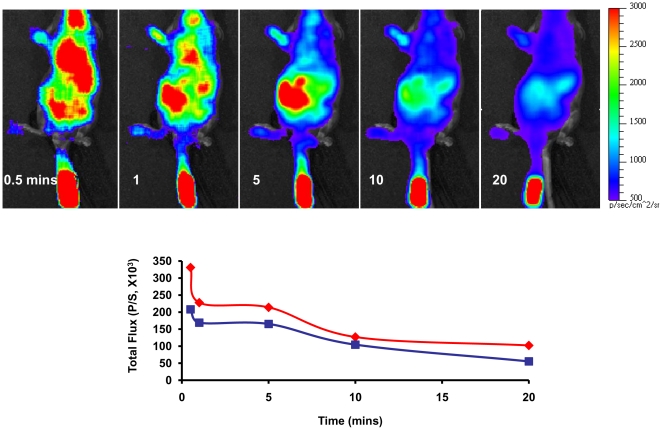
Imaging β-gal activity in transgenic 129S-Gt (ROSA) 26Sor/J mice mouse. Following IV injection of Galacto-Light Plus mixture (200 µl) light emission was observed with 60 s exposure time over 20 mins, with intense signal at point of injection in tail and time dependant varying signal from central organs (frontal view). Graph shows variation in signal intensity in tail near point of injection (blue) and separately for the mouse body (red).

Following IV injection of Galacto-Light PlusTM mixtures into 129S-Gt (ROSA)26Sor/J mice light emission was observed extensively throughout the body (Figure 3), though with somewhat lower intensity than for MCF7-*lacZ* tumor consistent with the lower β-gal activity (Figure S2 vs. S4). As an alternate strategy the substrate was administered subcutaneously (SC) on the back of the mouse, which generated very local, albeit far more intense signal, which showed maximum intensity after 5 mins and decreased over the following 30 mins (Figure S3). β-gal activity was also demonstrated in excised tissues by direct application of the mixture and expression was confirmed by X-gal staining of tissue surface (Figure S4).

## Discussion

We have demonstrated the ability to detect β-gal activity non-invasively using optical imaging *in vivo* following administration of Galacto-Light Plus™. BLI was successful in identifying *lacZ* versus WT tumors following either direct intratumoral or systemic intravenous administration of the chemiluminescent substrate together with reaction buffer, and accelerant. BLI also showed extensive light emission corresponding to β-gal expression throughout the body of black furry 129S-Gt(ROSA)26Sor/J mice following IV administration.

The value of any new technology must be placed in the context of existing methods or alternate approaches. Optical imaging is receiving much attention with dramatic innovations in reporter agents, applications and methods. New fluorescent materials reveal tumor locations and potentially surgical margins [26], dynamic bioluminescence reveals efficacy of vascular disrupting agents [27] and radionuclides may be detected by optical imaging [28]. Several crucial strengths are immediately apparent for BLI of chemiluminescent substrate. Intra venous administration of substrates avoids the constraints/requirements of knowing *a priori* where the expression will be observed, which confounds many existing *in vivo* imaging approaches to β-gal activity based on direct intra tumor injection of substrate. This potentially allows observation of deeper tumors without the need for needle access and potential tissue damage due to direct needle insertion into the tissue. Light emission avoids the background auto fluorescence, which handicaps fluorescent reporter molecule strategies. The commercial Galacto-Light Plus kit is designed for plate reader assays and includes four components: substrate, reaction buffer, accelerant buffer, and lysis buffer. Including all components provided greater light emission presumably because cell lysis releases intra cellular β-gal facilitating better enzyme substrate interaction. However, omitting the lysis buffer appears more satisfactory, particularly, for longitudinal studies *in vivo* and we have observed no apparent toxicity over four days following administration of the remaining mixture to mice.

Tumors expressing β-gal were detected, and extensive tissue radiance was observed in ROSA26 mice following IV administration of the substrate mixture. Direct injection into *lacZ* tumors gave even higher light emission, but SC injection in ROSA26 mice showed local light emission only, which appears quite different from bioluminescence (BLI) detection of luciferase expression. Others have shown that luciferin crosses physiological barriers (*e.g*., blood-brain and maternal-fetal [29]) and several groups have shown effective BLI following subcutaneous (SC), intraperitoneal (IP), intravenous (IV), or direct tissue injection of luciferin [30], [31], [32]. Unlike traditional luciferase-based BLI, signal intensity tended to decline quite rapidly after administering substrate, though light emission continued for many minutes. Selective detection was confirmed in excised tissues by *in situ* imaging and histology (Figure S2 and S4).

In comparison to NMR or nuclear imaging techniques, optical imaging is limited due to tissue light absorption and scattering. Maximum light emission was measured around 540 nm both in solution and minced β-gal expressing tumor tissue (Figure S1). This is a slightly shorter wavelength than the emission reported for the action of firefly luciferase on luciferin [33]. We note a major goal of bioluminescent and fluorescent imaging is development of longer wavelength emissions, and this may be feasible using wavelength shifters developed for CLI. Others have recently reported use of chemiluminescent substrates for *in vivo* imaging of mice, notably detection of myeloperoxidase based on IV infusion of luminol [34] and hydrogen peroxide based on peroxalate nanoparticles [35].

Here, we have demonstrated the ability to detect β-gal activity, but we note that other chemiluminescence enzyme detection kits are available and expect that alkaline phosphatase and neuraminidase detection could also be effective *in vivo*. We do note that the current reagents have been designed for well plate readers optimized in the blue-green visible range, whereas red to near infrared would be optimal for *in vivo* imaging and they could likely be optimized for *in vivo* applications. Importantly, use of chemiluminescent reporter agents adds a new approach to the armamentarium of the pre-clinical imaging scientist and will provide new opportunities for *in vivo* biochemistry, molecular biology, and therapy.

## Materials and Methods

### Cells

MCF7 wild type and stably transfected *lacZ* cell line: *E.coli lacZ* gene (from pSV-β-gal vector, Promega, Madison, WI) was inserted into high expression human cytomegalovirus (CMV) immediate-early enhancer/promoter vector phCMV (Gene Therapy Systems, San Diego, CA) giving a recombinant vector phCMV/lacZ, which was used to transfect human MCF7 wild type breast cancer cell (ATCC, Manassas, VA) using GenePORTER2 (Gene Therapy Systems, Genlantis, Inc., San Diego, CA), as described in detail previously ^18^. The highest β-gal expressing colony was selected using G418 (1000 µg/ml) and G418 (200 µg/ml) was included for routine culture.

### Imaging

Optical imaging was performed with a Caliper Xenogen IVIS® Spectrum and images were analyzed using Living Image 3.1 software (Caliper Life Sciences, Hopkinton, MA). Beta-galactosidase (Sigma, St. Louis, MO Cat #G2513-3KU: 1U) was evaluated with Galacto-Light PlusTM (Tropix, Bedford, MA) substrate in various solution combinations. The Galacto-Light PlusTM commercial kit includes four components: Galacton substrate (3-chloro-5-(5'-chloro-4-methoxyspiro[1,2-dioxetane-3,2'-tricyclo[3.3.1.13,7]decan]-4-yl)phenyl β-*D*-Galactopyranoside (Galacton Plus, T2118), Figure 1a), accelerant (diethanolamine in buffer containing EmeraldTM enhancer, T2081), reaction buffer (T2070), and lysis buffer. MCF7-WT and MCF7*-lacZ* cells (1x10^3^ to 1X10^6^ cells in 100 µl PBS) were placed in wells in a black clear bottom 96 well plate (Corning Company, Corning, NY) and 10 µl of various Galacton mixtures were added with or without the lysis buffer. Images were acquired in 5 to 120 s.

### *In vivo* imaging

Investigations were approved by the UT Southwestern Institutional Animal Care and Use Committee under APN #0464-07-32. MCF7-WT and -*lacZ* cells (1X10^6^) were implanted SC respectively in the left or right flanks of six female nude mice [19]. When tumors reached about 5 mm diameter, Galacto-Light Plus mixture was injected intravenously (100 µl) or intratumorally (50 µl comprising 30 µl substrate +10 µl accelerant +10 µl reaction buffer or 30 µl (20∶5∶50). Similarly, four 129S-Gt (ROSA)26Sor/J mice (The Jackson Laboratory, Bar Harbor, ME) were injected SC (with 25 µl mixture) or IV (100 µl or 200 µl mixture). The anesthetized (isoflurane (1.5%) in oxygen at 1.5 dm^3^/min) nude mice bearing MCF7-WT and –*lacZ* tumors and ROSA26 mice were observed using the IVIS® Spectrum. Images were acquired up to 180 mins after injection including dorsal and frontal views with various exposure times.

### *Ex vivo* imaging and X-gal staining

Tumors and organs were excised from mice after *in vivo* imaging and 30 µl Galacto-Light plus mixture was added dropwise onto the tissues. Imaging was performed immediately using the IVIS Spectrum with 30 s exposures. Organs were also stained with X-gal solution (1 mg/ml, Research Products International Corp., Mt. Prospect, IL) for 8 hrs and photographed.

### Histology

Tumors were excised after imaging and embedded in Tissue-Tek OCT (Miles Laboratory, Elkhart, IN) and frozen in liquid nitrogen. Cryostat sections were collected on gelatin-coated glass slides, and 8 µm sections stained with nuclear fast red (Sigma) and 1 mg/ml X-gal solution and with H & E (Sigma) individually.

### β-gal Assay

The β**-**gal activity of tumor cells and tissues in mice was measured using the β**-**gal assay kit (Promega) with yellow o-nitrophenyl β-*D*-galactopyranoside (ONPG). The extracted protein was quantified by a protein assay (Bio-Rad, Hercules, CA, USA) based on the Bradford method [36]. The enzyme activity is expressed as units/mg protein, where one unit corresponds to the hydrolysis of 1.0 µmol ONPG/min.

### Western blot

Protein was extracted from MCF7-WT and –*lacZ* tumors and other normal organs, and quantified using the Bradford method. Each well was loaded with 30 µg protein, separated by 10% SDS-PAGE (Nu-PAGE), and transferred to a polyvinylidene fluoride (PVDF) membrane. Primary monoclonal anti-β**-**gal antibody (Promega) and anti-actin antibody (Sigma) were used as probes at a dilution of 1∶5000, and reacting protein was detected using a horseradish peroxidase-conjugated secondary antibody and ECL detection (Amersham, Piscataway, NJ, USA).

## Supporting Information

Figure S1 Emission spectrum for Galacto-Light PlusTM reaction mixture with β-gal. A mixture of substrate (0.5 µl Galacto-Light Plus), accelerant buffer (5 µl) and reaction buffer (4.5 µl) was observed after addition of β-gal enzyme (2 U β-gal in 10 µl PBS) with various emission filters from 500 nm to 840 nm. Inset shows similar spectrum obtained when minced MCF7-lacZ tumor tissue was used in place of enzyme.
(0.54 MB TIF)

Figure S2 Verification of β-gal activity. a) Upper row sections from MCF7-lacZ tumor; Lower row from and MCF7-WT; (left) detection of β-gal based on X-gal staining and nuclear fast and (right) H&E staining. b) β-gal activity in tissues of mouse with MCF7-lacZ and -WT tumors determined using colorimetric assay. c) Protein expression based on Western blot confirming high activity of β-gal in lacZ tumor with about 10% background in MCF7-WT.
(1.22 MB TIF)

Figure S3 Imaging β-gal activity in transgenic 129S-Gt (ROSA)26Sor/J mouse following SC injection of substrate. Following SC injection of Galacto-Light Plus reaction mixture (10 µl) highly localized light emission was observed from the region of injection. The time dependent signal intensity curve shows maximum light emission after about 5 mins with decay over the next 30 mins. Signal was much more intense than following IV injection.
(0.33 MB TIF)

Figure S4 Detection of β-gal activity in organs of transgenic 129S-Gt (ROSA) 26Sor/J mouse by bioluminescence and β-gal staining. Left: BLI based on Galacto-Light Plus reveals lacZ expression *ex vivo* in various organs. Galacto-Light Plus mixture (25 µl) was injected into the tissue post mortem and detected with a 60 s exposure time. Right: Tissue surface staining after exposure to 1 mg/ml X-gal solution at 37°C for 8 hrs. Bottom: β-gal activity detected in organs using colorimetric assay.
(0.50 MB TIF)

## References

[1] Baker M (2010). Whole-animal imaging: The whole picture.. Nature.

[2] Weissleder R, Pittet MJ (2008). Imaging in the era of molecular oncology.. Nature.

[3] Jacob F, Monod J (1961). Genetic regulatory mechanisms in the synthesis of proteins.. J Mol Biol.

[4] Murakami T, Kobayashi E (2005). Color-engineered rats and luminescent LacZ imaging: a new platform to visualize biological processes.. J Biomed Opt.

[5] Olesen CEM, Yan Y-X, Liu B, Martin D, D'Eon B (2000). Novel methods for chemiluminescent detection of reporter enzymes.. Methods Enzymol.

[6] Eustice DC, Feldman PA, Colberg-Poley AM, Buckery RM, Neubauer RH (1991). A sensitive method for the detection of beta-galactosidase in transfected mammalian cells.. Biotechniques.

[7] James AL, Perry JD, Chilvers K, Robson IS, Armstrong L (2000). Alizarin-beta-D-galactoside: a new substrate for the detection of bacterial beta-galactosidase.. Letters Appl Microbiol.

[8] Chilvers KF, Perry JD, James AL, Reed RH (2001). Synthesis and evaluation of novel fluorogenic substrates for the detection of bacterial beta-galactosidase.. J Appl Microbiol.

[9] Masuda-Nishimura I, Fukuda S, Sano A, Kasai K, Tatsumi H (2000). Development of a rapid positive/absent test for coliforms using sensitive bioluminescence assay.. Lett Appl Microbiol.

[10] Li L, Zemp RJ, Lungu G, Stoica G, Wang LV (2007). Photoacoustic imaging of lacZ gene expression in vivo.. J Biomed Opt.

[11] Van Dort ME, Lee KC, Hamilton CA, Rehemtulla A, Ross BD (2008). Radiosynthesis and evaluation of 5-[125I]iodoindol-3-yl-beta-D-galactopyranoside as a beta-galactosidase imaging radioligand.. Mol Imaging.

[12] Celen S, Cleynhens J, Deroose C, de Groot T, Ibrahimi A (2009). Synthesis and biological evaluation of (11)C-labeled beta-galactosyl triazoles as potential PET tracers for in vivo LacZ reporter gene imaging.. Bioorg Med Chem.

[13] Lee KH, Byun SS, Choi JH, Paik JY, Choe YS (2004). Targeting of lacZ reporter gene expression with radioiodine-labelled phenylethyl-beta- d-thiogalactopyranoside.. Eur J Nucl Med Mol Imaging.

[14] Cui W, Otten P, Li Y, Koeneman K, Yu J (2004). A novel NMR approach to assessing gene transfection: 4-fluoro-2-nitrophenyl-β-*D*-galactopyranoside as a prototype reporter molecule for b-galactosidase.. Magn Reson Med.

[15] Kodibagkar VD, Yu J, Liu L, Hetherington HP, Mason RP (2006). Imaging b-galactosidase activity using ^19^F chemical shift imaging of LacZ gene-reporter molecule 2-fluoro-4-nitrophenol-β-*D*-galactopyranoside.. Magn Reson Imaging.

[16] Yu JX, Otten P, Ma Z, Cui W, Liu L (2004). A Novel NMR Platform for Detecting Gene Transfection: Synthesis and Evaluation of Fluorinated Phenyl β-*D*-Galactosides with Potential Application for Assessing LacZ Gene Expression.. Bioconj Chem.

[17] Kodibagkar VD, Yu J, Liu L, Hetherington HP, Mason RP (2006). Imaging beta-galactosidase activity using ^19^F chemical shift imaging of LacZ gene-reporter molecule 2-fluoro-4-nitrophenol-beta-D-galactopyranoside.. Magn Reson Imaging.

[18] Liu L, Kodibagkar VD, Yu J-X, Mason RP (2007). ^19^F-NMR detection of lacZ gene expression via the enzymic hydrolysis of 2-fluoro-4-nitrophenyl β-*D*-galactopyranoside *in vivo* in PC3 prostate tumor xenografts in the mouse.. FASEB J.

[19] Yu JX, Kodibagkar VD, Liu L, Mason RP (2008). A ^19^F NMR Approach using Reporter Molecule Pairs to Assess β-Galactosidase in Human Xenograft Tumors in Vivo.. NMR Biomed.

[20] Louie AY, Huber MM, Ahrens ET, Rothbacher U, Moats R (2000). In vivo visualization of gene expression using magnetic resonance imaging.. Nature Biotechnol.

[21] Cui W, Liu L, Kodibagkar VD, Mason RP (2010). S-Gal®, A Novel ^1^H MRI Reporter for β-Galactosidase..

[22] Wehrman TS, von Degenfeld G, Krutzik PO, Nolan GP, Blau HM (2006). Luminescent imaging of beta-galactosidase activity in living subjects using sequential reporter-enzyme luminescence.. Nat Methods.

[23] Chang YT, Cheng CM, Su YZ, Lee WT, Hsu JS (2007). Synthesis and characterization of a new bioactivated paramagnetic gadolinium(111) complex [Gd(DOTA-FPG)(H2O)] for tracing gene expression.. Bioconjugate Chemistry.

[24] Tung CH, Zeng Q, Shah K, Kim DE, Schellingerhout D (2004). In vivo imaging of beta-galactosidase activity using far red fluorescent switch.. Cancer Res.

[25] Zhang GJ, Chen TB, Connolly B, Lin SA, Hargreaves R (2009). In vivo optical imaging of LacZ expression using lacZ transgenic mice.. Assay Drug Dev Technol.

[26] Zhou H, Luby-Phelps K, Mickey BE, Habib AA, Mason RP (2009). Dynamic Near-Infrared Optical Imaging of 2-Deoxyglucose Uptake by Intracranial Glioma of Athymic Mice.. PLoS ONE.

[27] Zhao D, Richer E, Antich PP, Mason RP (2008). Antivascular effects of combretastatin A4 phosphate in breast cancer xenograft assessed using dynamic bioluminescence imaging (BLI) and confirmed by magnetic resonance imaging (MRI).. FASEB J.

[28] Liu H, Ren G, Miao Z, Zhang X, Tang X (2010). Molecular Optical Imaging with Radioactive Probes.. PLoS ONE.

[29] Thorne SH, Contag CH (2005). Using in vivo bioluminescence imaging to shed light on cancer biology.. Proc IEEE.

[30] Contero A, Richer E, Gondim A, Mason RP (2009). High-throughput quantitative bioluminescence imaging for assessing tumor burden.. Methods Mol Biol.

[31] Paroo Z, Bollinger RA, Braasch DA, Richer E, Corey DR (2004). Validating bioluminescence imaging as a high-throughput, quantitative modality for assessing tumor burden.. Molecular Imaging.

[32] Inoue Y, Kiryu S, Izawa K, Watanabe M, Tojo A (2009). Comparison of subcutaneous and intraperitoneal injection of *d*-luciferin for in vivo bioluminescence imaging.. European Journal of Nuclear Medicine and Molecular Imaging.

[33] Hui Z, Timothy CD, Olivier C, Flora K, Bradley WR (2005). Emission spectra of bioluminescent reporters and interaction with mammalian tissue determine the sensitivity of detection in vivo.. Journal of Biomedical Optics.

[34] Gross S, Gammon ST, Moss BL, Rauch D, Harding J (2009). Bioluminescence imaging of myeloperoxidase activity in vivo.. Nature Medicine.

[35] Lee D, Khaja S, Velasquez-Castano JC, Dasari M, Sun C (2007). In vivo imaging of hydrogen peroxide with chemiluminescent nanoparticles.. Nat Mater.

[36] Bradford MM (1976). A rapid and sensitive for the quantitation of microgram quantities of protein utilizing the principle of protein-dye binding.. Analyt Biochem.

