# Supplementary figures and images for: Imaging β-Galactosidase Activity in Human Tumor Xenografts and Transgenic Mice Using a Chemiluminescent Substrate
Source: PLoS One. 2010 Aug 6;5(8):e12024. doi: 10.1371/journal.pone.0012024 (PMC2917367; doi:10.1371/journal.pone.0012024)

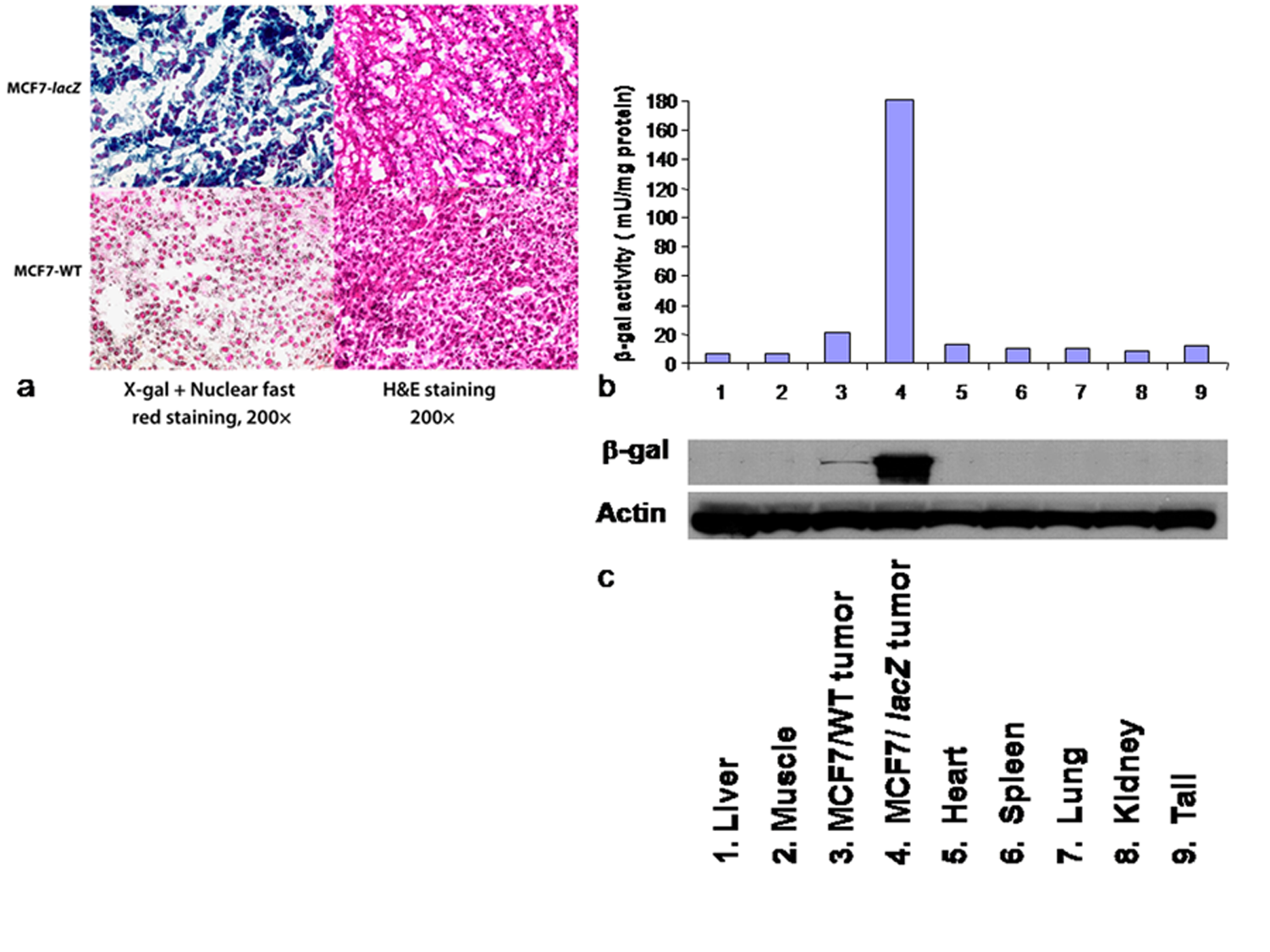

Supplement: Figure S2 — Verification of β-gal activity. a) Upper row sections from MCF7-lacZ tumor; Lower row from and MCF7-WT; (left) detection of β-gal based on X-gal staining and nuclear fast and (right) H&E staining. b) β-gal activity in tissues of mouse with MCF7-lacZ and -WT tumors determined using colorimetric assay. c) Protein expression based on Western blot confirming high activity of β-gal in lacZ tumor with about 10% background in MCF7-WT. (1.22 MB TIF) [file pone.0012024.s002.tif]

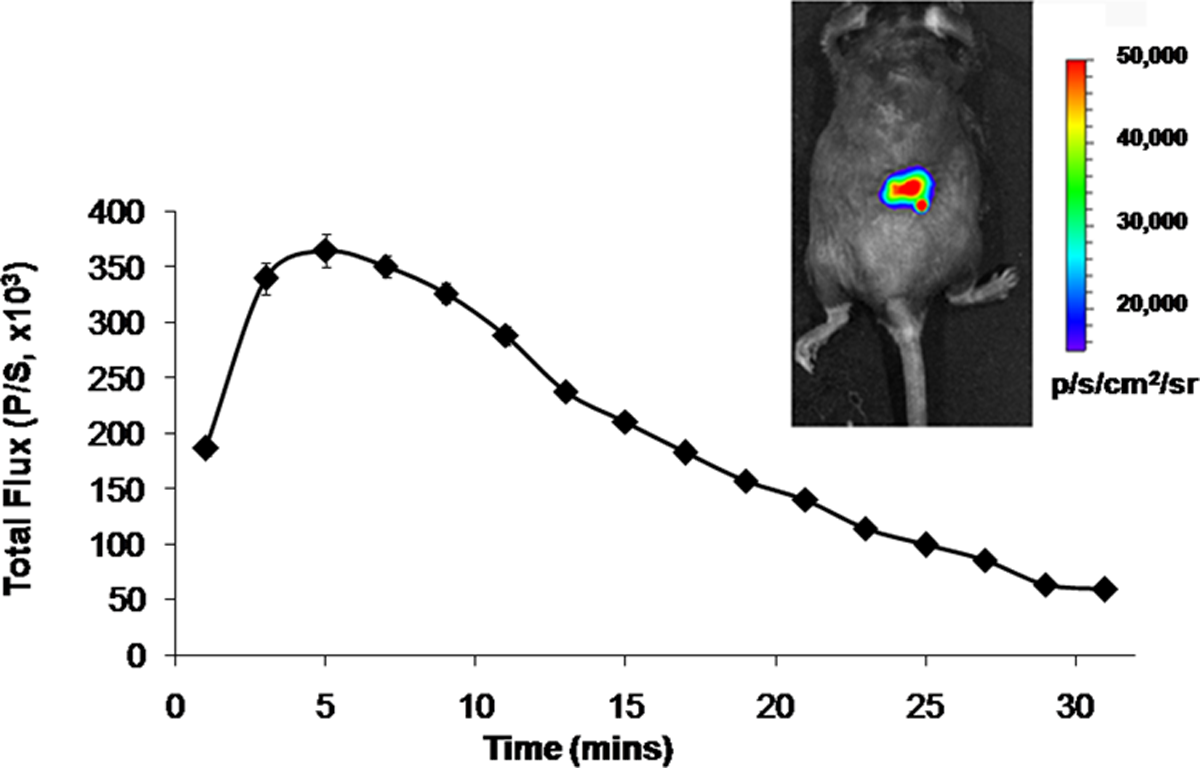

Supplement: Figure S3 — Imaging β-gal activity in transgenic 129S-Gt (ROSA)26Sor/J mouse following SC injection of substrate. Following SC injection of Galacto-Light Plus reaction mixture (10 µl) highly localized light emission was observed from the region of injection. The time dependent signal intensity curve shows maximum light emission after about 5 mins with decay over the next 30 mins. Signal was much more intense than following IV injection. (0.33 MB TIF) [file pone.0012024.s003.tif]

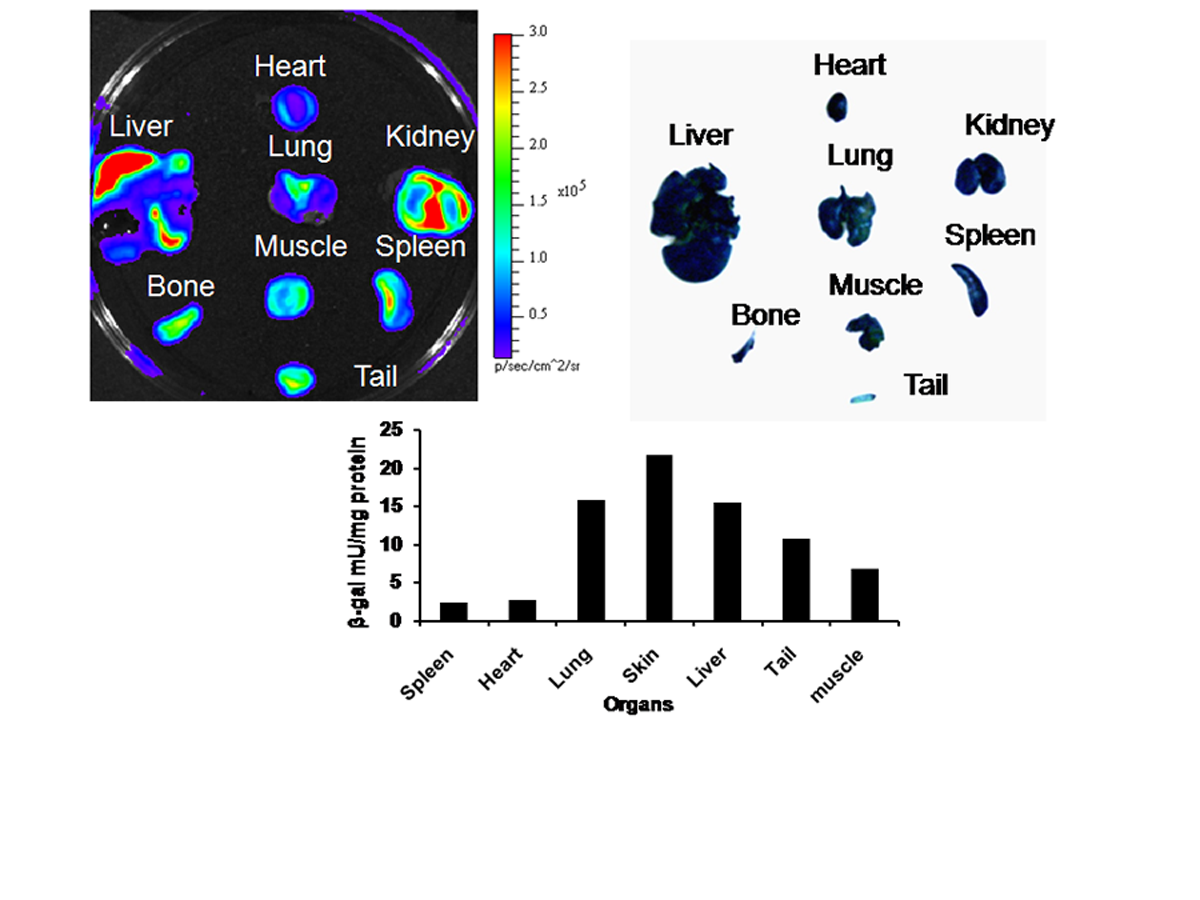

Supplement: Figure S4 — Detection of β-gal activity in organs of transgenic 129S-Gt (ROSA) 26Sor/J mouse by bioluminescence and β-gal staining. Left: BLI based on Galacto-Light Plus reveals lacZ expression ex vivo in various organs. Galacto-Light Plus mixture (25 µl) was injected into the tissue post mortem and detected with a 60 s exposure time. Right: Tissue surface staining after exposure to 1 mg/ml X-gal solution at 37°C for 8 hrs. Bottom: β-gal activity detected in organs using colorimetric assay. (0.50 MB TIF) [file pone.0012024.s004.tif]
